# Microneedle-Assisted Transdermal Delivery of Etanercept for Rheumatoid Arthritis Treatment

**DOI:** 10.3390/pharmaceutics11050235

**Published:** 2019-05-15

**Authors:** Jian Cao, Nan Zhang, Ziyi Wang, Jingjing Su, Jing Yang, Jiabing Han, Yongxing Zhao

**Affiliations:** 1 Department of Pharmaceutics, School of Pharmaceutical Sciences, Zhengzhou University, Zhengzhou 450001, China; justin1140@163.com (J.C.); nanzhang@zzu.edu.cn (N.Z.); wangziyia315@163.com (Z.W.); sujingjing0530@163.com (J.S.); jingyang0021@163.com (J.Y.); hanjiabing9283@163.com (J.H.); 2 Key Laboratory of Targeting Therapy and Diagnosis for Critical Diseases, Zhengzhou 450001, China; 3 Key Laboratory of Advanced Pharmaceutical Technology, Ministry of Education of China, Zhengzhou 450001, China

**Keywords:** microneedle, etanercept, rheumatoid arthritis, drug delivery, hyaluronic acid

## Abstract

Rheumatoid arthritis (RA) is a complicated autoimmune disease. The clinical applications of etanercept (EN), a TNF-α inhibitor, can efficiently halt the development of RA. EN is mainly administrated by subcutaneous injection, which may cause low compliance, side effects, and infection risk. In this study, a hyaluronic acid crosslinked microneedle system (MN) was constructed as the transdermal alternative to deliver EN. We describe the formulation, fabrication, characterization, and transdermal insertion study of MN. In vitro bioactivity of EN was conducted and analyzed by dynamic light scattering and circular dichroism spectrum. In vivo evaluation of MN was studied on adjuvant-induced arthritis mice. The MN possessed sufficient mechanical strength, good biocompatibility, little influence on the bioactivity of EN, and high anti-inflammatory efficacy. This work represents a successful example of delivering macromolecule therapeutic treatment by MN for RA treatment. The transdermal delivery of EN by MN offers a new treatment option for RA patients.

## 1. Introduction

Rheumatoid arthritis (RA) is a systemic autoimmune disease with chronic inflammation and joint swelling, stiffness, and erosion [1]. Severe RA causes functional disability, organ failure, infection, and even death [2]. Although the etiology of RA remains unclear, it is acknowledged that proinflammatory cytokine tumor necrosis factor-α (TNF-α) plays a critical role in the pathogenesis of RA [3,4]. In clinic, anti-TNF therapy is considered a credible therapeutic approach and is found to regulate processes like angiogenesis, secretion of cytokine and chemokine, bone turnover, etc. [1]. To date, five TNF inhibitors (infliximab, adalimumab, certolizumab pegol, golimumab, and etanercept) have been approved in the market. Etanercept (EN), a fusion protein consisting of the recombinant human TNF-receptor p75 monomer fused with the Fc domain of human IgG1, has been widely used due to its high efficacy and safety [5]. However, like other protein drugs, the administration route of EN is limited to subcutaneous injection (SC), which needs additional reconstitution and injection operation. Furthermore, long-term SC will lead to pain, infection risk, and low compliance [6,7]. These disadvantages urge researchers to develop a new administration route with high bioequivalence, low risk, and improved compliance.

The skin is the largest organ of the human body with approximately 2 m^2^ of surface area, providing a broad area for the potential administration of drugs [6]. The painless nature of the transdermal application of medicine offers improved acceptance and compliance. In addition, drug absorption in the skin avoids the first-pass hepatic metabolism [8]. However, the barrier function of stratum corneum dramatically reduces the efficiency and efficacy of transdermal delivery [9]. Only some lipophilic and small molecular (less than 500 Da) drugs can be absorbed by passive permeation in a low efficiency [10]. Many technologies such as iontophoresis [11], sonophoresis [12], and electroporation [13] have been developed to enhance the penetration of the skin. However, these methods need to use additional instruments and may damage the skin.

Microneedle (MN), which consists of many microscopic needles with lengths ranging from 50 to 900 μm, has overcome the challenges of the skin barrier [14]. MN directly pierces the stratum corneum barrier and generates transient microchannels to deliver the encapsulated drug without stimulating nerves and damaging blood vessels [15]. Therefore, MN is a cost-efficient and minimally invasive transdermal delivery system. Many groups have reported that MN is a credible option to deliver macromolecular drugs like vaccines [16], nucleic acid [17], and hormones [18] with little influence on their bioactivity. Among all types of MN, the dissolvable MN shows superiority to deliver a large dose of the drug [19]. In addition, it is easy for self-administration after it is applied on the skin, as the drug is released from dissolvable MN and absorbed automatically without any additional operations. 

**Figure 1:**
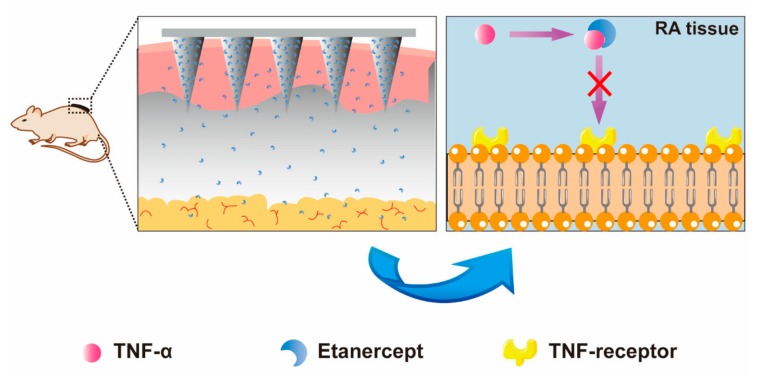
Schematic illustration of the concept. After the application of microneedle system (MN) on the dorsal skin of mice, etanercept (EN) is released from MN and absorbed by tissue blood capillary. In the arthritis tissue, EN takes effects by blocking the binding of TNF-α and TNF receptor.

Herein, we developed a dissolvable hyaluronic acid (HA) MN with high biocompatibility, bioequivalence, and enhanced compliance to deliver EN (Figure 1). The crosslink process under the Ultra Violet (UV) light enhanced the mechanical strength. Our work encompasses the preparation, characterization, and skin insertion study to ensure effective delivery into the skin. Dynamic light scattering (DLS) and circular dichroism spectrum (CD) were used to examine the in vitro bioactivity of EN after the UV curing. Furthermore, we investigated the in vivo efficacy of MN administration on adjuvant-induced arthritis (AIA) mice model by assessing paw swelling, clinical score of hind limbs, TNF-α and IL-6 level in serum.

## 2. Materials and Methods

### 2.1. Materials

EN was purchased from Shanghai CP Guojian Pharmaceutical Co., Ltd. (Shanghai, China). HA (molecular weight 300kDa) was obtained from Freda Biochem Co., Ltd. (Shandong, China). Methacrylic anhydride (MA), 2-Hydroxy-4′-(2-hydroxyethoxy)-2-methylpropiophenone (I 2959), *N*,*N*′-methylenebisacrylamide (MBA) and Complete Freund’s Adjuvant (CFA) were obtained from SIGMA-ALDRICH (Merk, Darmstadt, Germany). Sulforhodamine B (SRB) was purchased from Yuanye Biotechnology Co., Ltd. (Shanghai, China). Trypan blue was purchased from Beyotime Biotechnology (Shanghai, China). TNF-α ELISA kit was purchased from Solarbio Life Science (Beijing, China) and IL-6 ELISA kit was purchased from MultiSciences (Hangzhou, China). All chemicals for preparation and analysis were obtained from the commercial source and used as received. Ultrapure water (18 MΩ cm; GenPure, Thermo Fisher Scientific, Waltham, MA, USA) was used for all solution preparations.

### 2.2. Synthesis and Characterization of Acrylate- Modified HA (mHA)

mHA was synthesized following a previously reported method [20]. Briefly, HA (1.0 g) was dissolved in ultrapure water (50 mL) at 4 °C under continuous stirring overnight. *N*,*N*-dimethylformamide (DMF, 50 mL) was added dropwise and mixed completely. Then MA (1.19 mL) was added, while maintaining the pH between 8 and 9 using NaOH solution (0.5 M). The reaction was kept at 4 °C for 6 h. After that, NaCl was added to obtain a concentration of 0.5 M. mHA was precipitated using acetone with a water/acetone ratio of 1/3 (*v*/*v*) and washed with ethanol thrice. The product was re-dissolved and dialyzed against ultrapure water for 48 h to remove residual organic reagents. Finally, mHA product was obtained by freeze drying.

To prove the successful synthesis of mHA, mHA was dissolved in deuterium oxide at a concentration of 15 mg/mL and measured by nuclear magnetic resonance spectrum (NMR, AVANCE III HD 400M, Bruker, Beijing, China). The degree of methacrylation was calculated by comparing the areas under proton peak at 5.74 ppm (CH_2_ of methacrylate) to the peak at 1.99 ppm (CH_3_CO of HA).

### 2.3. Fabrication and Characterization of MN

MN was fabricated by the micromoulding method [21]. In brief, EN (2.5 mg/mL), MBA (25 mg/mL), and I 2959 (0.5 mg/mL) were dissolved in water. mHA (40 mg/mL) was added and swelled completely. Then, the solution was centrifugated at a speed of 7000 rpm for 5 min to remove bubbles. The solution was pipetted on the surface of PDMS micromold (Blueacre Technology Ltd., Dublin, Ireland) and put in a vacuum oven (600 mmHg) for 5 min to allow the solution to flow into the cavities. Afterwards, the micromolds were centrifugated with a swinging bucket rotor at a speed of 2500 rpm for 6 min to compact the tips. These procedures were repeated three times. To fabricate the base layer, the micromold was surrounded by the silver adhesive taper. There was 1 mL of HA solution (40 mg/mL) added into the taper reservoir followed by overnight drying at 30 °C in a vacuum oven (760 mmHg). After the complete desiccation, MN was carefully detached from the mold and tailored into patch form. To enhance the mechanical property, MN was crosslinked by exposing to UV light (wavelength: 365 nm, intensity: 1900 mW/cm^2^, Futansi Electronic Technology Co., Ltd., Shanghai, China) for 2 min. The morphology of MN array was detected using an optical microscope (DM 3000, Leica, Frankfurt, Germany) and a focused ion beam scanning electron microscopy (SEM, Auriga FIB, Carl Zeiss AG, Heidenheim, Germany).

### 2.4. Mechanical Strength Measurement

The mechanical strength of MN was measured by force-displacement analysis, using texture analyzers (TA.XT plusC, Stable Micro Systems, London, UK). MN was tailored into a 5 × 6 array and immobilized on the bottom steel plate. The initial distance between tips of MN and the top sensor was set at 2 mm. The speed of the top sensor toward the MN was 0.01 mm/s. The force and displacement were recorded 200 times per second.

### 2.5. Skin Penetration Study

SPF Swiss mice were obtained from the Laboratory Animal Center of Zhengzhou University (Zhengzhou, China). All animal care and experiments were approved by the animal ethical committee of Zhengzhou University (Project identification code: 20170310), according to the requirement of the National Act on the Use of Experimental Animals (China). Mice were acclimated for 1 week before the study.

Before the experiment, mice were anesthetized and hair on the dorsal skin was removed by hair clipper and depilatory cream. Mice were then kept for 24 h for skin recovery. MN was applied to the skin of mouse with a thumb for 5 min and subsequently removed. The mouse was euthanized immediately and the skin sample was separated carefully and imaged using digital camera. To prove the effective insertion, the skin was stained by trypan blue solution (3%, *w*/*v*) for 5 min. An alcohol swab removed the residual solution and the skin was imaged under optical microscopy. In another experiment, the applied skin tissue was fixed with 10% formalin for 48 h. Then the tissue was embedded in paraffin, sectioned at the penetration site and stained with hematoxylin and eosin (H&E). The obtained sections were imaged under optical microscopy.

### 2.6. MN Dissolution and Skin Recovery

MN array was applied to the dorsal skin of hair-removed mice with thumb and immobilized by medical adhesive tape (Nexcare ^®^, 3M, Maplewood, MN, USA) for 0.5 h, 1 h, 1.5 h, 2 h, respectively. At each predetermined time, MN was detached and imaged under microscopy to observe the dissolution of needles. To study the skin recovery, MN was applied to the skin for 10 min and removed. Afterward, the MN treated skin was imaged by a digital camera every 15 min until complete recovery.

### 2.7. Drug Loading of MN

EN content was measured according to the previous study [22]. EN was mixed with SRB at a ratio of 4/1 (*w*/*w*) and dissolved in water. The fluorescent intensity of SRB-EN solution ranging from 31.25 to 0.4882 μg/mL was measured using a plate reader (PerkinElmer EnSpire, Waltham, MA, USA). The excitation and emission wavelengths were set at 570 and 600 nm. To determine the EN content encapsulated in MN, the SRB-EN (2.5 mg/mL) was encapsulated in MN as described above. MN was incubation with 2 mL PBS buffer in a 6 well plate on a shaker. After MN was completely dissolved, the fluorescent intensity of the sample solution was measured and the content of EN in MN was calculated according to the standard curve.

### 2.8. In Vitro Bioactivity of EN After UV Curing

EN was homogeneously spread on the filter paper and exposed to UV light for 0 s, 60 s, 120 s, and 180 s, respectively. The particle size and zeta potential of EN [23] were measured using a Zetasizer Nano ZS90 (Malvern, UK). Briefly, 1 mL of each dissolved sample (0.3 mg/mL) was measured using disposable sizing cuvette for hydrodynamic size and disposable capillary cell for zeta potential. In another experiment, the secondary structure was detected by CD measurement [24] with a Chirascan Circular Dichroism Spectrometer (Applied Photophysics, Leatherhead, UK). In brief, after background scanning, the ultrapure water was measured as a reference to obtain a baseline. Then the EN sample (0.1 mg/mL) was loaded into the quartz cell (Hellma Analytics, Munich, Germany) with a light path of 1 mm. The scanning of each sample was ranged from 190 to 260 nm with a resolution of 1 nm and repeated thrice. The obtained spectrum was processed by Pro-Data Chirascan and Pro-Data Viewer software.

### 2.9. Preparation of AIA Model

Mice were injected subcutaneously at the hind paw with 80 μL CFA (1 mg/mL). There was 40 μL of CFA injected on the same paw on the day 7 after the initial immunization. Mice were observed and evaluated with the clinical scoring system every day [25]. Two weeks after the first immunization, mice with a score 3 or 4 were selected and divided into 3 groups randomly: saline-treated mice (SA), EN treated using SC mice (eSC) and EN treated using MN mice (eMN). 

### 2.10. Therapeutic Effect of MN on AIA Mice

The inflamed mice were treated with saline (30 μL), EN (1 mg/kg) by SC and MN every two days. MN was applied on the dorsal skin of mice and immobilized by medical adhesive tape for 2 h for absolute dissolution of the needles. Normal mice were also used as a control and treated with saline (30 μL). Paw thickness, paw swelling, paw flexibility were recorded every day and clinical scores were calculated. Body weight was measured every two days before the treatment. The mice were sacrificed after 10-day treatments and blood samples were collected. TNF-α and IL-6 level in serum were determined by ELISA kit. The joints of mice were collected and sliced for histologic examination. The sliced samples were stained with hematoxylin and eosin (H&E) and observed under optical microscopy (DM1000, Leica, Frankfurt, Germany).

### 2.11. Statistical Analysis

The data were performed by Origin software (Version 9.5.1, OriginLab Corporation, Northampton, MA, USA, 2018). Statistical significance was analyzed by GraphPad Prism software (Version 5.01, GraphPad Software, Inc., La Jolla, CA, USA, 2007) with one-way ANOVA among multiple groups or student’s test between two groups. The presented data (mean ± SD) resulted from at least three independent experiments (*n* ≥ 3). *p* value < 0.05 was considered significant.

## 3. Results

### 3.1. Fabrication and Characterization of the Crosslinked MN

**Figure 2:**
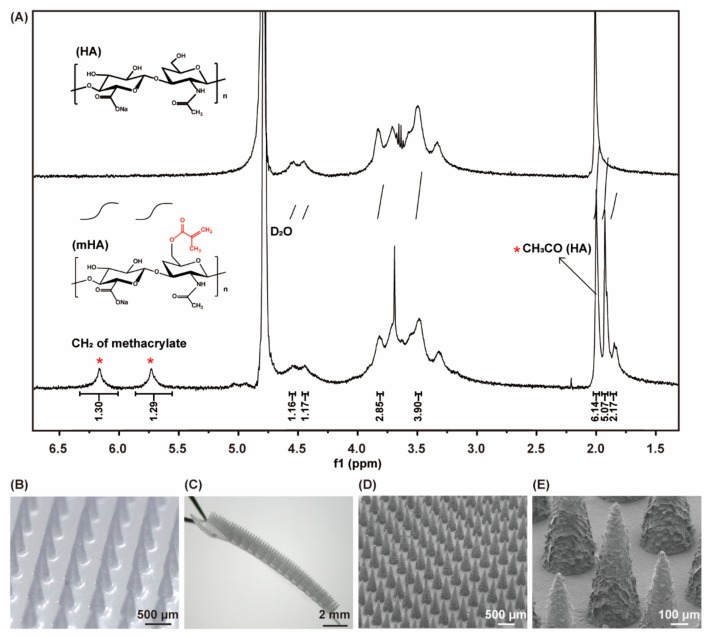
(**A**) Nuclear magnetic resonance (^1^H-NMR) spectrum of HA and mHA; the microscope images (**B**,**C**) and scanning electron microscopy (SEM) images (**D**,**E**) of MN.

HA was methacrylated by reacting with MA in the mixture of water and DMF. The proton peaks at 6.17 and 5.74 ppm represent methacrylate protons, suggesting the successful modification. The degree of methacrylation was approximately 20% and was sufficient for crosslink (Figure 2A). The MN array was fabricated by micromouding method. The array was composed of 15 × 15 conical needles with 20 μm diameter at tip, 300 μm diameter at the base, and 800 μm in height (Figure 2B–E). The base made by HA had great flexibility and toughness, which is suitable for MN application on irregular skin. The SEM images (Figure 2D,E) showed the ragged surface of the needles because MBA and crosslink process would result in a rough surface after drying [26].

### 3.2. MN Insertion Study

**Figure 3:**
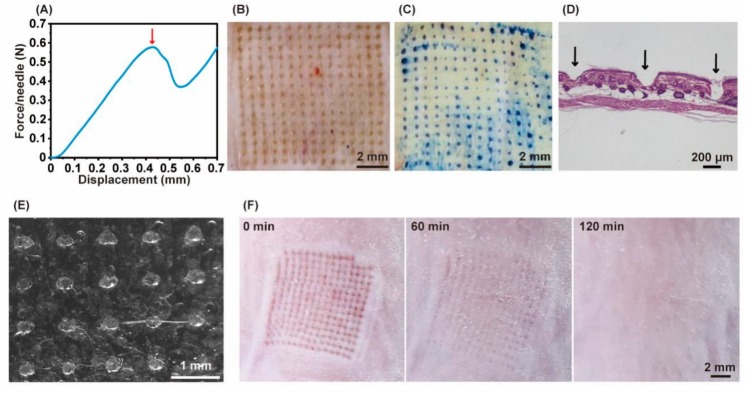
(**A**) Examination of the failure force per needle; the separated mouse dorsum skin (**B**) and trypan blue stained skin image (**C**); (**D**) H&E-stained cross-section of inserted skin by MN; (**E**) MN image after application to the mouse skin for 90 min; (**F**) skin recovery images at 0 min, 60 min, and 120 min after MN treatment.

The failure force can be detected by force-displacement analysis. Once the MN fractures, a sudden drop will show on the curve. The failure force of MN was 0.58 N per needle, which is sufficient for skin insertion (Figure 3A). After applying to mouse skin, micropores were shown on the insertion site of the skin (Figure 3B). Trypan blue selectively stained viable epidermis thus demonstrated the puncture marks of MN (Figure 3C). Furthermore, the microchannel (Figure 3D) caused by MN was evidenced by H&E staining cross-section. MN penetrated to a depth of approximately 200 μm. All these results confirmed the effective insertion. The high hydrophilicity of HA enables almost complete dissolution of MN in the skin after 90 min, the residual base of needles was shown in Figure 3E. The inserted skin recovered quickly after the removal of MN and recovered completely after 120 min (Figure 3F).

### 3.3. In Vitro Bioactivity Study of EN and Drug Content in MN

**Figure 4:**
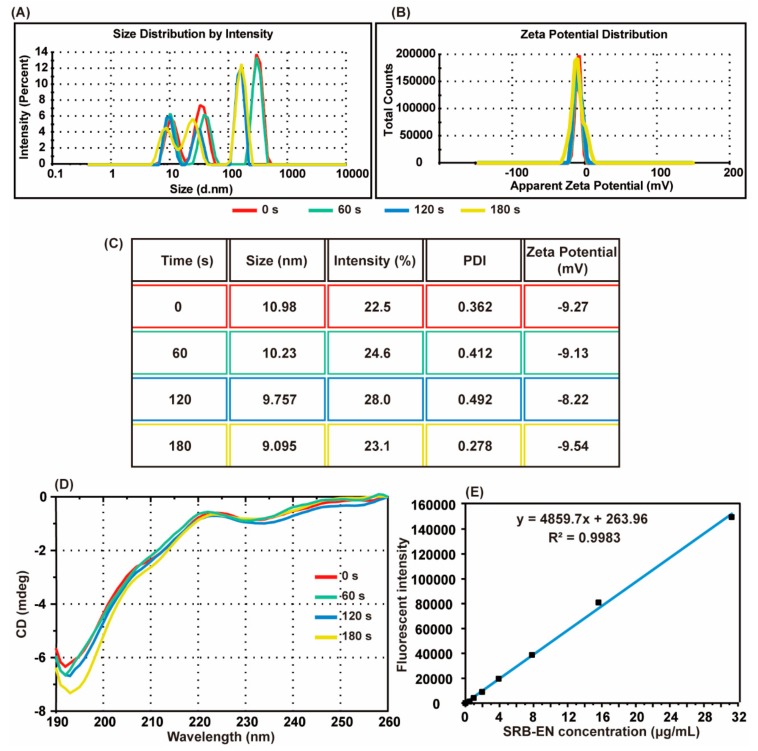
Size distribution by intensity (**A**) and zeta potential distribution (**B**) of EN after the exposure to Ultra Violet (UV) light for 0 s, 60 s, 120 s, and 180 s; (**C**) the table of size, intensity, polydispersity index, and zeta potential of the listed samples; (**D**) circular dichroism spectrum of listed samples; (**E**) a standard curve of the fluorescent intensity for SRB-EN mixture.

To assess the in vitro bioactivity of EN during UV curing, EN was first exposed to UV light for 0 s, 60 s, 120 s, and 180 s, respectively. DLS was used to assess the aggregates between EN and drug excipients in the nanometer size. Figure 4A shows the size distribution of the sample solution. The size of EN was approximately 10.10 nm with a polydispersity index (PDI) less than 0.5 (Figure 4C), which is slightly larger than previously reported 7.1 nm for EN [27]. This may be caused by adsorption of sugar on the surface of EN. The size distribution by intensity was very sensitive to the existence of aggregation [27]. The treated-EN with different UV curing time showed little difference in size intensity (Figure 4A). Zeta potential value means the electrical repulsion among the surrounding of protein. A moderate value of absolute zeta potential suggests good colloidal stability of protein samples. The UV curing did not exhibit significant affection on the zeta potential of EN (Figure 4B). Therefore, UV has little influence on EN aggregation.

Circular dichroism (CD) was widely used to detect the secondary structure changes of protein samples [24]. If UV light destroys the secondary structure of EN, a change of the CD spectrum should be expected. The CD wavelength profile was similar between different samples, with a deep peak at 192 nm and a shallow peak at 223 nm (Figure 4D). Notably, when the curing time is 180s, the deep peak was slightly shifted to 194 nm, which may indicate the beginning of the unfolding of EN.

SRB is a strong and sensitive fluorescent agent, which can bind to the amino of protein. Therefore, the amount of EN in MN can be measured referring to the fluorescence intensity of SRB-EN mixture with an *R*^2^ = 0.9983 (Figure 4E). The average of EN per MN array was about 42.72 ± 5.81 μg, which is sufficient for in vivo treatment evaluation. And the dosage can be adjusted by the tailor of MN.

### 3.4. Therapeutic Effect on AIA Mice

**Figure 5:**
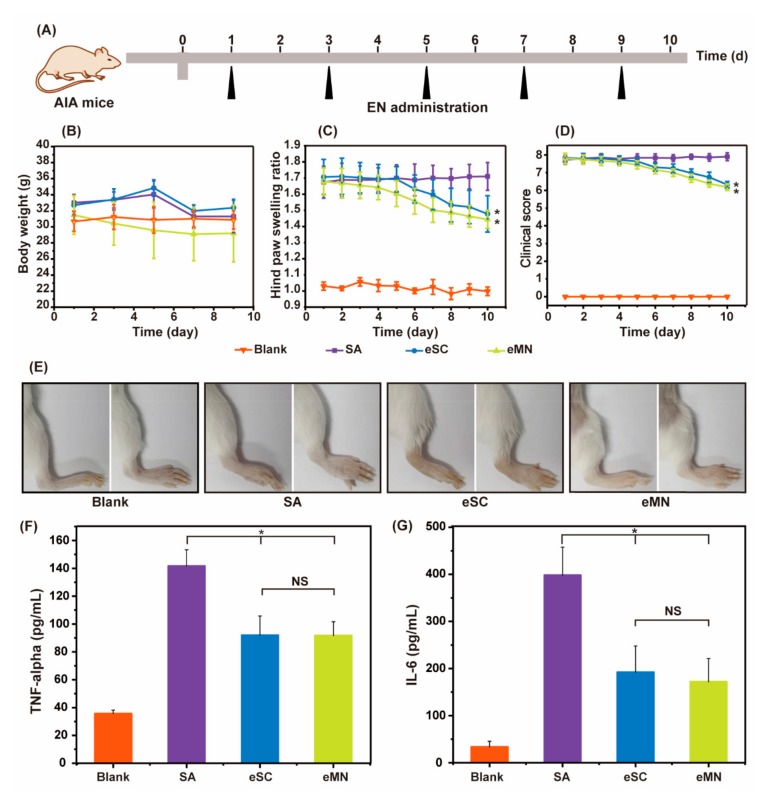
(**A**) The timeline of the animal experiment; (**B**) Body weight, (**C**) paw swelling ratio and (**D**) clinical score of AIA mice during 10 days of treatment using the listed treatment. (**E**) The frontal and profile images of hind paw of each group. (**F**) the TNF-α and IL-6 (**G**) concentration of serum after listed treatment. ^*^ *p* < 0.05 vs. SA. NS represents no significance.

**Figure 6:**
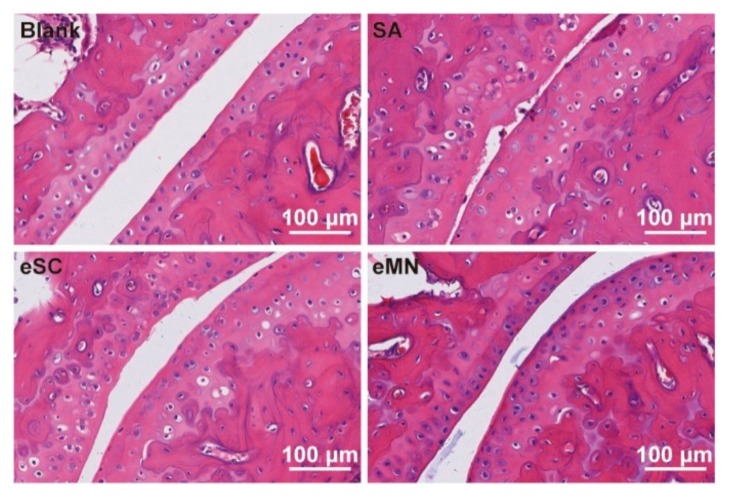
Representative H&E stained images of ankle joints from normal mice and AIA mice treated with SA, eSC, and eMN.

The therapeutic effect of MN was investigated on AIA mice. The mice were treated with saline (SA), EN by SC (eSC), and EN by MN (eMN) for 10 days according to the timeline shown in Figure 5A. Anesthesia and hair removal operation may cause a slight decrease in body weight of MN group initially and maintained a weight of 30 g afterwards (Figure 5B). The paw swelling and clinic score were monitored every day. Mice with untreated arthritis maintained a high clinic score and paw thickness. In the EN-treated groups, though the swelling of the hind limb or symptom was not completely eliminated, the paw swelling ratio (Figure 5C) in eSC group and eMN group were reduced from 1.70 to 1.48 and 1.68 to 1.44 in 10 days, respectively, suggesting an anti-inflammatory effect. It seems that EN takes effect more quickly by MN treatment. However, there is no significance in the therapeutic effect between these two groups. The clinic score results were consistent with the change of swelling ratio (Figure 5D). The frontal and profile images of hind paw at 10 days (Figure 5E) showed the visible benefit from EN treatment. The TNF-α and IL-6 level in serum increased dramatically after injection of CFA. After treatment, concentrations of TNF-α and IL-6 in serum were reduced in eSC and eMN groups (Figure 5F,G). Pathological slides showed a poor joint structure in the SA group. By contrast, eSC and eMN effectively protect the joint from erosion. (Figure 6) In summary, MN showed good bioequivalence to the classical SC administration.

## 4. Discussion

RA is an autoimmune chronic disease that dramatically reduces the quality of life for patients [28]. Over the past years, RA-related inflammation cannot be completely eliminated, but well controlled by TNF inhibitor [29]. However, a majority of TNF inhibitors are administrated by injection either via SC or intravenous injection. Long-term SC injection leads to side effects and even serious infection. It also increases the psychological stress of patients. In this study, MN, a new transdermal drug delivery system, has been used to address limitations of hypodermic injection. MN has all the advantages of injection and transdermal administration by piercing the skin barrier without stimulating nerves and blood vessels [30]. Among all types of MN, dissolvable MN is made of soluble materials, generally biocompatible polymers or polysaccharides [31]. Previous studies have proposed that dissolvable MN presents numerous advantages on drug loading, fabrication, and safety [15,19,32]. Importantly, it generates no biohazardous sharp waste and eliminates needle re-use after administration [33]. HA was used as the material of MN for that it is a natural glycosaminoglycan and the major component of the extracellular matrix in the skin [34]. Furthermore, the HA molecule is easy to modify [20] to enhance the property of MN. In summary, MN made by HA is a promising transdermal drug delivery system of EN.

Photopolymerization is a widely-used method to promote the stiffness of hydrogels. This reaction is initiated by the decomposition of a photoinitiator upon UV or visible light. The crosslink process is fast and clean in a mild condition compared with chemical crosslink [35]. Considering the strength of classical HA-MN may be not enough to insert into the skin to a certain depth [36]. Gu’s group introduced UV crosslink into the MN preparation to enhance the strength of MN [26,37]. Accordingly, in our study, DMF was added as a co-solvent in the modification of HA to control the degree of methacrylation to 20% in a faster way. The mechanical strength measured by texture analyzer was about 580 mN per needle, which is sufficient for skin insertion. We then did the skin insertion study on mice for that mice skin have a similar structure to the human skin and easy to get. The results showed approximately 200 μm depth of penetration. The viscoelastic nature of the skin and the rapid softening of needles can explain this. Regardless, we have confirmed the effective insertion of MN and ensured future applications on human subjects. Due to the high biocompatibility and hydrophilicity of HA, MN presents a quick dissolution and skin recovery.

The bioactivity of EN is susceptible to the environmental factor. In clinic, the product of EN is mainly in freeze-dry powder form and must be stored at 4 °C. During our fabrication process, the temperature is one susceptible influence factor. In Giulbudagian’s study [38], CD was performed to detect the structural stability with different temperature. The thermal denaturation of EN was found to occur at temperature ≥ 45 °C. Therefore, they suggested that the operating temperature should be below 37 °C. In our study, the temperature of drying was controlled at 30 °C and it only takes approximately 24 h to finish complete desiccation in vacuum condition. Another destructive factor to EN is UV light. Bryant’s study showed that I 2959 is the most cytocompatible UV initial compound. Even after exposing to UV light for 10 min, nearly all NIH/3T3 fibroblasts survived [39]. It seems UV curing has no damage to protein in a short period. However, the influence of UV curing on EN is still unclear. We used DLS to detect the aggregation of EN and CD to detect the structure change according to Kim’s studies [23,27]. DLS results showed that UV curing time had little influence on the hydrodynamic size and zeta potential of EN. The CD spectrum showed that EN had no unfolding in 120 s and may have little unfolding in 180 s during the UV curing. Therefore, the UV curing time in our fabrication was set at 120 s.

The inflammatory cytokines in serum are significantly associated with the pathogenesis of RA [40]. TNF-α and IL-6 are key cytokines to evaluate the therapeutic effect. Interestingly, in comparison to the eSC group, eMN showed similar efficacy in paw swelling, clinical score, cytokines, and joint erosion. It has been proved that the MN-based delivery system may enable rapid lymphatic uptake of protein drugs. By NIR fluorescent imaging, Harvey et al. [41] also found that MN induced regional lymphatic drainage. The lymph-driven uptake may contribute to the rapid absorption of the macromolecule. Courtenay et al. [42] have proved that the MN delivery system posed sustained pharmacokinetics of bevacizumab. In addition, a number of in vivo studies showed that the MN had good efficacy on health conditions such as diabetes [18], cancer [43], obesity [44], etc. These studies may explain the similar therapeutic effect to the classical SC and confirm the promising potential of MN. Recently, many groups have explored new fabrication technologies [45] and the application of large MN patches has been successfully evaluated on human volunteers [46,47,48]. In our study, MN with high biocompatibility and bioequivalence is easy to fabricate. The principle of the micromoulding method is simple, and has a bright opportunity for mass production. We deeply believe that these advances would make it possible for commercialization and clinical application of MN in the future.

## 5. Conclusions

In the present study, we have developed a crosslinked dissolvable MN system, as a carrier to deliver EN. It represents a successful example to deliver macromolecule by MN for RA treatment. The MN with high mechanical strength could easily pierce the skin, dissolve in body fluid and deliver EN to the blood capillary. Due to the high biocompatibility and hydrophilicity, MN shows the complete dissolution and quick skin discovery. Our mild fabrication condition ensures the little influence on the bioactivity of EN. For AIA mice treatment, MN shows good bioequivalence to SC administration and higher compliance. In conclusion, MN presents a great potential for future application of transdermal delivery of EN.
